# Full-term pregnancy with retroperitoneal giant mucinous cyst: A case report and literature review

**DOI:** 10.1097/MD.0000000000036979

**Published:** 2024-03-08

**Authors:** Jiao Wen, Yun Zhao, Fei Tang, Wenxing Cheng, Jing Peng, Qianyi Li, Haotian Pan, Hao Li, Lei Chen

**Affiliations:** a Department of Obstetrics, Maternal and Child Health Hospital of Hubei Province, Tongji Medical College, Huazhong University of Science and Technology, Wuhan, China; b Wuhan University of Science and Technology, Wuhan, China; c Hubei University of Medicine, Shiyan, China; d Huazhong University of Science and Technology, Wuhan, China.

**Keywords:** cystadenoma, full-term pregnancy, retroperitoneal mass

## Abstract

**Rationale:**

Retroperitoneal benign cysts during pregnancy are extremely rare and often remain asymptomatic until they attain a very large size. Diagnosis typically relies on a pathological tissue biopsy. The decision to pursue 1-step or 2-step surgical treatment should be tailored to each individual case rather than generalized.

**Patient concerns:**

This case report presents the unique scenario of a pregnant woman with a confirmed pregnancy complicated by a large retroperitoneal cyst. The patient had a retroperitoneal cyst during her initial pregnancy, which went undetected during the first cesarean section. However, it was identified during her second pregnancy by which time it had grown to 13.0 cm × 15.0 cm × 25.0 cm, and extended from the liver margin to right ovarian pelvic infundibulopelvic ligament. Consequently, it was removed smoothly during her second cesarean section.

**Diagnoses:**

Postoperative pathology results indicated a massive retroperitoneal mucinous cystadenoma.

**Interventions:**

The giant retroperitoneal cyst was smoothly excised during the second cesarean delivery for 1-step surgical treatment.

**Outcomes:**

Under the combined spinal and epidural anesthesia, a live female infant was delivered at 38 3/7 gestational weeks and the neonatal weight was 3200g. Under general anesthesia with endotracheal intubation, the giant retroperitoneal cyst was excised smoothly without complications.

**Lessons:**

The findings of this case report contribute to the understanding of the diagnostic modalities, surgical approaches and postoperative considerations of giant retroperitoneal cysts associated with pregnancy.

## 1. Introduction

A primary retroperitoneal cyst is an extremely rare developmental cyst. The incidence is estimated at 0.4 to 0.9 per 100,000 cases.^[1–4]^ Typically, primary retroperitoneal cysts do not exhibit symptoms, and they are often only identified and diagnosed as such when they reach a significant size and exert pressure on vital abdominal organs. Retroperitoneal cysts can be classified into different categories based on their origins, which may include traumatic, infectious, degenerative, and neoplastic. Common types of retroperitoneal cysts encompass ovarian cysts,^[5,6]^ lymphocysts,^[7]^ intestinal cysts,^[8]^ cystic teratoma, mesothelioma,^[9]^ and degenerative neurofibroma. The first reported case of a pregnancy combined with a retroperitoneal cyst was in 1965, and it was considered one of the most unusual clinical manifestations during pregnancy.^[10]^ So far, there are still no standardized criteria for its clinical characteristics, diagnosis, management, and the timing of neonatal delivery. In this report, we describe a case involving a large retroperitoneal cyst discovered during mid-trimester pregnancy, which was surgically removed during a cesarean section. Additionally, we conducted a systematic review of the presentation, imaging, and types of surgical interventions for pregnancies with retroperitoneal cysts.

## 2. Case report

**Figure 1.**
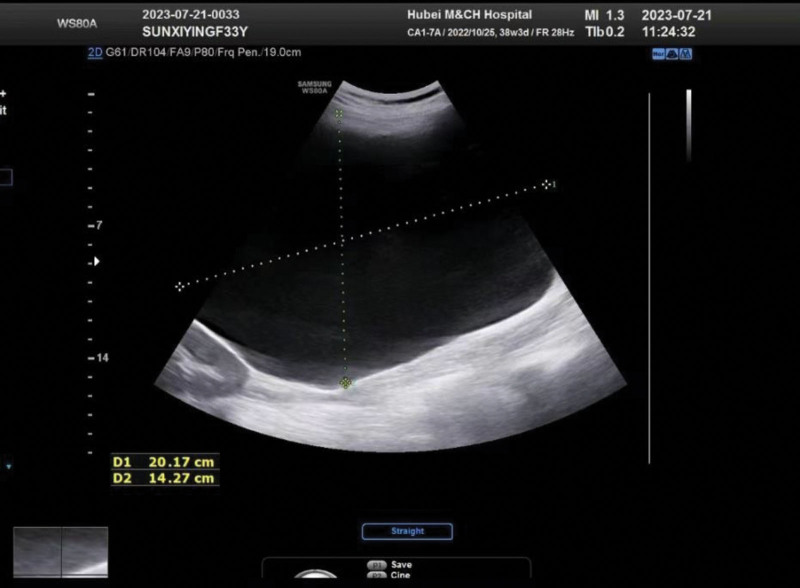
Huge retroperitoneal cystic mass shown on ultrasound.

We present a case of a 33-year-old pregnant woman who was admitted to our hospital on July 24th, 2023, at 38 6/7 weeks of pregnancy with a history of a prior cesarean delivery. She had regular menstrual cycles, with her last menstrual period on October 25th, 2022, indicating an expected due date of August 1st, 2023. The patient underwent regular prenatal checkups, totaling 10 visits. Throughout her pregnancy, she did not experience any discomforts such as dizziness, fatigue, or lower abdominal pain. On December 19th, 2022, at 7 6/7 gestational weeks, an early intrauterine pregnancy without apparent abnormalities in the adnexal area was found by ultrasound. On April 6th, 2023, at 23 2/7 gestational weeks, a singleton live fetus and a hypoechoic mass measuring about 11.8 cm × 13.9 cm × 18.3 cm in the right abdominal area without distinct septation was found by ultrasound. On April 14th, 2023, at 24 3/7 gestational weeks, a cystic lesion with long T1 and T2 signals in the right abdominal area, measuring approximately 14.1 cm (anteroposterior) × 12.1 cm (transverse) × 20.3 cm (superior-inferior) with clear borders, homogeneous signal, exerting pressure on adjacent structures, and being closer to the right ovary was found by MRI,as shown in Table 1.On July 21st, 2023, at 38 3/7 gestational weeks, a singleton live fetus with a hypoechoic mass measuring 12.7 cm × 14.3 cm × 24.5 cm in the patient right abdominal area without distinct septation was found by ultrasound as shown in Figure 1. She had previously undergone a cesarean delivery in October 2014 at another hospital, at 39 gestational weeks, due to a maternal request for a cesarean delivery. At that time, an adnexal cyst approximately 5.0 cm × 6.0 cm was identified through ultrasound on admission, but no mass was identified in the uterus or adnexal area. She did not undergo any further ultrasound or MRI examinations until the current pregnancy. The tumor markers of Carbohydrate antigen 19-9(CA19-9), Carcinoembryonic antigen, Carbohydrate antigen 125(CA125) were within normal ranges both during pregnancy and on admission, as shown in Table 2. An elective cesarean delivery was performed due to her previous cesarean delivery and the presence of a large abdominal mass.

**Table 1:** Ultrasound and MRI suggestive of tumor size changes during this pregnancy.

	2023.04.06	2023.04.14	2023.05.15	2023.07.21
Ultrasound				
Region	Right side of the abdomen		Right side of the abdomen	Right side of the abdomen
Size (cm)	13.9*18.4*11.8		19.5*12.3*11.3	24.5*12.7*14.3
Characteristic	No echo, no light band separation		No echo, no light band separation	No echo, no light band separation
MRI				
Region		Right side of the abdomen		
Size (cm)		14.1*12.1*20.3		
Characteristic		Cystic long T1, T2 signaling shadow with well-defined borders		

MRI = magnetic resonance imaging.

**Table 2:** Changes in tumor markers during this pregnancy.

Date	2023.04.18	2023.07.24
Alpha-fetoprotein	312.11 ng/mL↑	616.32 ng/mL↑
CA-125	2.51 U/mL	17.60 U/mL
CA-199	19.23 U/mL	2.33 U/mL
CA15-3	/	17.60 U/mL
Carcinoembryonic antigen	1.34 ng/mL	1.02 ng/mL

**Figure 2.**
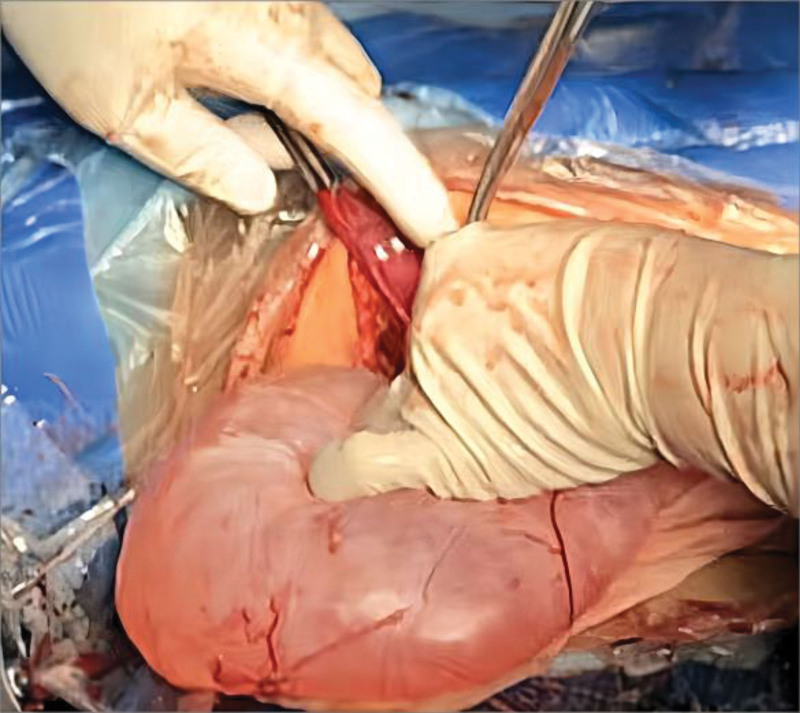
Cyst after draining 2/3 of the cystic fluid intraoperatively.

Under the combined spinal and epidural anesthesia, a live female infant was delivered in the left occiput anterior position, with Apgar scores of 9 at 1 minute and 10 at 5 minutes. The neonatal weight was 3200 g. Following delivery, the uterus and adnexa were examined within the abdomen, and no abnormalities were detected. However, during subsequent abdominal exploration, a large cystic mass was identified behind the right peritoneum. This mass had a smooth surface and extended from the liver margin to right ovarian pelvic infundibulopelvic ligament, measuring approximately 13.0 cm × 15.0 cm × 25.0 cm, as shown in Figure 2.

A multidisciplinary team (MDT) was established during the surgery, involving obstetrician-gynecologists, general surgeons and anesthesiologists. Two treatment options were considered. The first option involved the immediate removal of the retroperitoneal cyst, while the second option entailed a staged surgery scheduled for 2 months after childbirth. Considering that the mass was a single-cyst tumor, and the surgical procedure was expected to be relatively simple, we chose to proceed with the immediate removal of the cyst.

**Figure 3.**
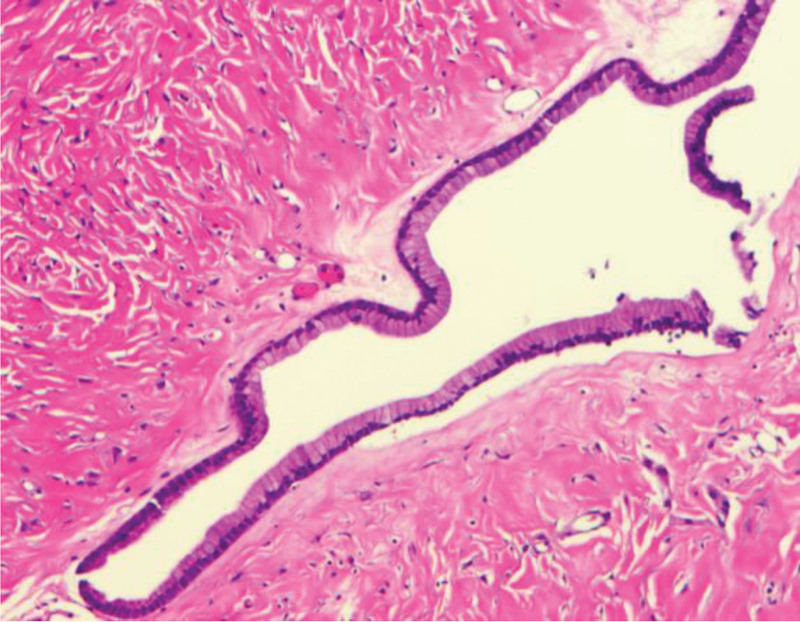
Mucinous epithelium of mucinous cystadenoma shown microscopically.

Under general anesthesia with endotracheal intubation, it was challenging to displace the uterus to the side, and allowing for limited exposure of the cyst location. An incision was made in the posterior peritoneum. The cyst was situated in close proximity to the abdominal aorta, vena cava, and their branches, surrounded by a complex network of blood vessels and nerves. As a result, we made the decision to open and drain a portion of the cyst fluid until its volume had been reduced by 2/3. This opening was sutured, and the cyst was meticulously separated using a combination of blunt and sharp dissection techniques. The surgical area was thoroughly irrigated with a saline solution, effectively controlling any bleeding. The cyst was carefully separated using a combination of blunt and sharp dissection techniques. After this, the patient underwent tumor excision. A preliminary pathological examination suggested the mass was a benign mucinous cyst with calcification, as shown in Figure 3.

Subsequently, the abdomen incision was closed, with one drainage tube left in the pelvic cavity for postoperative drainage. The patient was administered cefoperazone-sulbactam (1g q12h × 2d) during and after the surgery, and then transferred to the intensive care unit postoperatively. On the day of the surgery, an ultrasound revealed a localized deep vein thrombosis in the left calf, with an inner diameter of approximately 0.8cm and a low-echo structure measuring about 1.7 cm × 0.6 cm. Stagnant blood flow was also observed in the right calf intramuscular vein, which had an inner diameter of about 0.5 cm. A pulmonary artery computed tomography angiography (CTA) indicated the presence of multiple emboli in both pulmonary artery branches. Subsequently, anticoagulation therapy was initiated post-surgery, involving the use of low molecular weight heparin (Enoxaparin 4100 iu Q12h*4) and Warfarin (2mg, Qd). The abdominal drainage tube was removed on the fourth day after surgery, and the patient was discharged 9 days after surgery. A routine postoperative pathological examination revealed the presence of a mucinous cystadenoma with multifocal calcifications in the cyst wall.

## 3. Discussion

Tumors in the retroperitoneum are relatively uncommon. They can be either malignant or benign, with malignancies being more prevalent^.[11]^ Malignant retroperitoneal tumors may include primary liposarcoma,^[12]^ epithelioid sarcoma,^[13]^ malignant mesenchymal tumors, or metastatic tumors, primarily from the spread of germ cell tumors.^[14]^ On the other hand, benign retroperitoneal tumors are exceptionally rare. They may include lymphatic cyst,^[15]^ benign neurogenic tumors,^[16–18]^ cellular angiofibroma,^[19]^ fibromatosis,^[1,19–21]^ retroperitoneal ectopic pregnancy,^[22]^ and fetal retroperitoneal solid mature teratoma^.,[23]^ as detailed in Table 3. Furthermore, mullerian cysts are typically observed in the ovaries and pancreas.^[32]^ When located in the retroperitoneum, these cysts can be mistaken for other retroperitoneal cysts, such as cystic lymphangioma and cystic mesothelioma of the peritoneum, due to nonspecific clinical and radiologic presentations.^[33]^ In our study, we report a large retroperitoneal cyst that was diagnosed as mucinous cystadenoma with multifocal calcifications in the cyst wall through histopathological analysis.

**Table 3:** Reported cases of primary retroperitoneal cystadenoma of pregnancy at home and abroad from 2000 to 2023.

	Discovery time(w)	symptom	Imaging (Ultrasound/MRI)	Tumor marker	Mode of operation	Postoperative examination
Sagili H, Acín-Gá^[24]^;Acín-Gándara D^[25]^;Hanhan HM^[26]^;Wang X^[27]^	8–20	Asymptomatic/Severe nausea, vomiting, lower back pain, fever	Huge retroperitoneal mass, pushing the bowel to 1 side/Displacement and compression of kidneys, liver, and inferior vena cava	Normal	Gestation (Percutaneous puncture conservative treatment)/Postpartum exploratory laparotomy	Retroperitoneal mucinous cystadenoma
Kashima K^[28]^;Berczi C^[29]^;	26–29	Asymptomatic	Left abdomen/ the pelvis contains a solid mass containing wall nodules with smooth, thin borders	Normal/CA19-9rise	Exploratory laparotomy (Gestation)	Primary retroperitoneal mucinous cystadenocarcinoma
Ramírez Daniel L^[16]^; AllenR^[17]^;Aragón-Mendoza RL^[18]^	2–33	Asymptomatic/lumbar scoliosis	Large, solid, heterogeneous retroperitoneal mass with irregular morphology suspected to be tumor soft tissue	Normal	Exploratory laparotomy (Postpartum)	Retroperitoneal ganglioneuroma
Ibraheim M^[20]^;Bertini R^[11]^;A. Mori^[21]^;Abe H^[19]^	8–25	Pain in the right lower back and abdomen/Bowel obstruction during pregnancy	A large (solid) well-defined mass can be seen retroperitoneally	Normal	Gestation/Postpartum Exploratory laparotomy	Fibroma
Ikeda T^[15]^	30	/	20*7cm retroperitoneal mass with fluid	/	Drainage, laparoscopic resection after delivery	Lymphatic cyst
Joshi U^[30]^	19	Severe epigastric pain with vomiting	A large mass with clear boundary, fat content and uneven echo can be seen on the right posterior peritoneum	Normal CA125	Exploratory laparotomy (Postpartum)	Mature teratoma
Hakamada K*^[31]^	25	Severe upper abdominal pain and vomiting	The retroperitoneal area is characterized by a large oval, unevenly enhanced mass with abdominal hematoma	/	/	/

CA125 = carbohydrate antigen 125, CA199 = carbohydrate antigen 19-9, MRI = magnetic resonance imaging.

* In this case, the maternal condition worsened due to late diagnosis and delayed treatment, which eventually led to the death of the pregnant woman and the fetal death in utero without an autopsy.

Diagnosing retroperitoneal tumors can be challenging due to their rarity and atypical clinical features, leading to potential misdiagnoses.^[12,19,32]^ Geetha SD et al^[32]^ reported a case of a retroperitoneal mucinous cystadenocarcinoma in a 54-year-old female presenting with right flank pain. Imaging revealed an 8.6 × 7.9 cm mass on the anterior surface of the lower pole of the right kidney, initially suspected as renal cell carcinoma. Serum tumor markers, including CA 19-9 and carcinoembryonic antigen were within normal limits, and CA125 was elevated. Similarly, Tani A et al^[12]^ reported a 78-year-old women with a 35-cm-diameter solid tumor in the peritoneal cavity. Elevated levels of CA125 and HE4 raised suspicions of ovarian malignancy. Unfortunately, she passed away 11 months after surgery due to disease progression. In our case, the patient was initially diagnosed with an ovarian cyst (5.0 cm × 6.0 cm) during her first pregnancy via ultrasound. However, during her cesarean delivery, no cyst was found in the uterus or adnexa. Nine years later, a large cyst was discovered that grew rapidly from 11.8 cm × 13.9 cm × 18.3 cm at 23 2/7 gestational weeks to 13.0 cm × 15.0 cm × 25 cm by the time of her second cesarean delivery. Our findings align with previous studies. Several factors may have contributed to the initial misdiagnosis in this case: The extreme rarity of retroperitoneal mucinous cysts and the lack of specific clinical features make them easily misdiagnosed as ovarian masses.^[33,34]^ During the first cesarean delivery, the cyst was relatively small and wasn’t examined during the surgery, leading to the assumption that it might have been a physiological ovarian cyst. Consequently, there was no regular postoperative follow-up regarding the cyst. In the early stages of the second pregnancy, due to its deep retroperitoneal location, the mass remained undetected, posing identification challenges. In the second and late trimester of pregnancy, the cyst was misdiagnosed as a large benign ovarian cyst because of its proximity to the right ovary, as observed in ultrasound and MRI scans, coupled with normal tumor marker levels.

In our surgical approach, the primary focus of discussion within our MDT team was whether to proceed with a 1-step surgery involving cesarean delivery combined with the resection of the retroperitoneal mass or to opt for a 2-step approach, where cesarean delivery would be performed first, followed by the resection of the retroperitoneal mass. Several key factors influenced our decision. We recognized that if the retroperitoneal mass continued to grow, it posed the risk of rupture or torsion at any moment, necessitating emergency surgery. At the same time, performing retroperitoneal mass resection during a cesarean section carried the potential of requiring a second operation due to the unclear nature of the mass, which could be challenging to fully address during the initial surgery. Performing retroperitoneal mass resection simultaneously with a cesarean section carries a high risk of damaging the internal iliac vessels, external iliac vessels and their branches, which can result in a massive retroperitoneal hematoma, then creating a critical and life-threatening situation. In this particular case, given the high likelihood of a benign tumor as indicated by ultrasound, MRI, tumor markers, and rapid intraoperative examination, a decision was made to perform both a cesarean section and tumor resection for 1-step procedure. The surgical procedure proceeded smoothly, with minimal intraoperative bleeding. Based on this experience, we recommend that the decision regarding the effectiveness of 1-step or 2-step surgical treatment should be made on a case-by-case basis rather than generalized.^[19]^

In our case, due to the large scope of the operation, anti-infection treatment with cezolin sodium was used after the operation. One day following the surgery, a pulmonary CTA examination detected the presence of pulmonary embolism, leading to the initiation of anticoagulant therapy with both warfarin and low molecular weight heparin. Considering the possible causes of thrombosis in this patient, several factors were taken into account: Firstly, hormonal changes during pregnancy can increase blood clotting factors, and the enlarged uterus and cysts may exert pressure on the vena cava, potentially leading to the development of venous thrombosis. Secondly, the extensive 2-step surgical procedure carried a risk of blood vessel damage during the operation. Lastly, postoperative rehabilitation often involves extended bed rest to promote wound healing, but prolonged immobility can cause blood to stagnate in the veins, increasing the risk of thrombosis.

## 4. Conclusion

During pregnancy, benign retroperitoneal tumors are extremely rare and usually remain asymptomatic until they reach a significant size. This case exemplifies a misdiagnosed retroperitoneal cyst initially mistaken for an ovarian mass, even with the advancements in CT, MRI, and tumor markers. The accurate diagnosis hinges on a pathological tissue biopsy. The decision to pursue a 1-step or 2-step surgical treatment should be tailored to individual cases rather than generalizing an approach.

## Acknowledgments

The authors thank the obstetricians and radiologists for the diagnosis and treatment of pregnant women.

## Author contributions

**Investigation:** Wen Cheng, Jing Peng, Qianyi Li, Haotian Pan, Hao Li.

**Writing – original draft:** Jiao Wen.

**Writing – review & editing:** Yun Zhao, Fei Tang.
